# Immediate and delayed effects of invented writing intervention in preschool

**DOI:** 10.1007/s11145-016-9646-8

**Published:** 2016-04-22

**Authors:** Hilde Hofslundsengen, Bente Eriksen Hagtvet, Jan-Eric Gustafsson

**Affiliations:** 1 Faculty of Teacher Education and Sport, Sogn og Fjordane University College, Box 133, 6851 Sogndal, Norway; 2 Department of Special Needs, University of Oslo, Oslo, Norway; 3 Department of Education and Special Education, University of Gothenburg, Gothenburg, Sweden

**Keywords:** Invented writing, Intervention, Early literacy, Invented spelling, Preschool practice

## Abstract

This study examined the effects of a 10 week invented writing program with five-year-old preschoolers (mean age 5.7 years) on their immediate post intervention literacy skills and also the facilitative effects of the intervention on the subsequent learning to read during the first 6 months of schooling. The study included 105 children (54 girls) from 12 preschools in Norway. The preschools were randomly assigned to the experimental group with the invented writing program, or the control group with the ordinary program offered to preschoolers. The classroom-based programs (40 sessions) were conducted by the children’s regular teachers. The children’s emergent literacy skills were evaluated using a pre-test, a post-test and a follow-up test 6 months later, and the data were analyzed using latent autoregressive models. The results showed that the invented writing group performed significantly better than the control group on the post-test for the measures of phoneme awareness (*d* = .54), spelling (*d* = .65) and word reading (*d* = .36). Additionally, indirect effects were observed on the delayed follow-up tests on phoneme awareness (*d* = .45), spelling (*d* = .48) and word reading (*d* = .26). In conclusion, we argue that invented writing appeared to smooth the progress of emergent literacy skills in preschool, including the subsequent reading development in school. Contextualized in a semi-consistent orthography and a preschool tradition that does not encourage the learning of written language skills, the findings add to our knowledge of how children learn to write and read.

## Introduction

Many preschool children actively explore the oral-written language relationships by spontaneously writing down oral sounds (words, syllables and phonemes) in creative, yet systematic, combination with letter naming; for example RUDF (are you deaf?), GNYS AT WRK (genius at work) or KAM (come) (Bissex, 1980; Clarke, 1988). This phenomenon may be labeled *invented writing* or *emergent writing* (Puranik & Lonigan, 2011; Whitehurst & Lonigan, 1998). If phoneme-graphemes are specifically in focus the phenomenon has often more narrowly been termed *invented spelling* (Chomsky, 1971; Read, 1971); however, the terms are also used interchangeably. The term *invented writing* refers to the written products of young children who are exploring and discovering the sound-text-relationships during their writing. It may include script elements such as scribbling, logos and letter-sound connections. The current study makes use of this often spontaneously driven activity of young children in an intervention program involving Norwegian five-year-olds.

Intervention studies of invented writing are scarce compared to other domains of literacy such as phonological awareness and letter knowledge. Moreover, the intervention studies that do exist have most often been carried out in irregular orthographies (e.g., English and French) with a focus on the training of spelling skills in a culture which emphasizes the importance of learning to read and spell during the time of the training. They have been conducted either by parents in the children’s homes (Aram & Levin, 2001), or by researchers (Levin & Aram, 2013; Martins, Salvador, Albuquerque, & Silva, 2014; Ouellette & Sénéchal, 2008; Rieben, Ntamakiliro, Gonthier, & Fayol, 2005; Sénéchal, Ouellette, Pagan, & Lever, 2012). Moreover, with one exception (Ouellette, Sénéchal, & Hayley, 2013), the studies have been short in time span, with pre-post designs covering preschool only. The current study expands this knowledge base. It focused on invented writing as a child-driven explorative activity, individually and in groups; it was conducted in Norwegian, which has a semi-consistent orthography; and it was conducted within a preschool context that places little emphasis on encouraging the children’s written language skills before schooling. The intervention program was furthermore carried out in the children’s preschool by their teachers, making it a more naturalistic study in line with Clarke (1988); it lasted for 10 weeks and the program effect was assessed both immediately after the intervention and with a longitudinal follow-up after half a year in school. More specifically, our aim was to investigate the effect of a 10-week invented writing program in preschool on phoneme awareness, spelling and word reading in preschool and in early schooling.

Children’s preschool literacy skills are well-known predictors of reading and writing development in school (National Early Literacy Panel, 2008). Children with poor early literacy skills are more likely to struggle with formal literacy learning in schools than children with well-developed early literacy skills. During the last decades, increased awareness of these relations has led to a growing interest among researchers and politicians in how preschool education can support the emergent literacy skills of preschool children. In a literate culture, most preschoolers take an interest in the written language; for example, they scribble, ask about the names of letters, study the sound structure of words and spontaneously try to find relevant letters to write simple words and names. This has been documented in numerous developmental studies (see Aram & Levin, 2001; Clay, 1975; Ferreiro & Teberosky, 1982; Hagtvet, 1988, 2010; Korsgaard, Vitger, & Hannibal, 2011; Liberg, 1993; Pontecorvo & Orsolini, 1996; Read, 1971; Tolchinsky, 2004; Treiman, 1993). Although developmentally elucidating, much of the research interest in preschool children’s writing prior to the new millennium was descriptive and anecdotal. Only fairly recent intervention studies have shown that children’s writing and reading skills may be facilitated by invented spelling programs in preschool (Levin & Aram, 2013; Martins, Albuquerque, Salvador, & Silva, 2013; Martins et al., 2014; Martins & Silva, 2006; Ouellette et al., 2013; Ouellette & Sénéchal, 2008; Rieben et al., 2005; Sénéchal et al., 2012). The few, mainly small scale intervention studies that do exist conclude that invented spelling stands out as a promising facilitator of emergent literacy skills.

It is well known that phonemic awareness is a crucial prerequisite skill in learning to read and spell (for example, Bradley & Bryant, 1983; Hatcher, Hulme, & Ellis, 1994; Lundberg, Frost, & Petersen, 1988) and studies of invented spelling training suggest that the exploration of sounds in words when spelling quite powerfully strengthens children’s phoneme awareness (Levin & Aram, 2013; Martins & Silva, 2006; Ouellette et al., 2013; Ouellette & Sénéchal, 2008; Sénéchal et al., 2012). In previous studies by Ouellette and Sénéchal (2008), Sénéchal et al. (2012), Ouellette et al. (2013), the invented spelling intervention group performed as well as the control group receiving phoneme awareness training on post-test phonological awareness skills. In addition, the children receiving the invented spelling program outperformed the controls on a learning-to-read task (i.e., the children were taught to read 10 words which were used in a recall trial), suggesting that the invented spelling training adds something more to the children’s emergent literacy skills than phonological awareness training in combination with letter knowledge training. A potential explanation for these added effects according to the authors is that the manipulation of phoneme-grapheme connection during writing made the children more meta-cognitively aware.

The invented spelling training has also shown significant effects on spelling measures (Levin & Aram 2013; Martins et al., 2014; Ouellette & Sénéchal, 2008; Sénéchal et al., 2012). Even children with low-phoneme awareness appeared to benefit from invented spelling training in a randomized control trial (RCT) by Sénéchal et al. (2012). All the participating children in this study had low phoneme awareness at pre-test, but at post-test the children receiving invented spelling training spelled novel words in more phonologically sophisticated ways than did the controls.

Reading and spelling skills are often considered closely connected because they draw on the same alphabetic knowledge base and develop in related stages (Ehri, 2000); however, the effect of invented writing training on reading skills is not clear, particularly with regard to whether such training actually affects reading of new words or just facilitates skills underpinning reading like phoneme awareness and letter knowledge. Only the studies by Martins et al. (2013, 2014) observed significant effects of invented spelling training on reading new words. However, the reading measure used included only short words (i.e., 2–4 letters), which could have meant that the task was not very challenging for the children. According to Martins et al. (2013) the positive effect of invented spelling training on reading in their study could have been due to Portuguese being considered to have a more consistent orthography than English and French. Hence type of orthography could affect the ease with which new words are read.

Taken together, the reviewed studies indicate that invented spelling intervention could positively influence phoneme awareness, spelling and early reading skills, but the number of studies is limited, and the findings are inconsistent with regard to its effect on reading skills. The purpose of the current study was to investigate the effect of an invented writing program broader than the previous ones, using a variety of activities that invited the children to explore sound-text relationship via writing. Similarly to the previous invented spelling interventions we welcomed the children’s explorative writing of the letter-phoneme relations, but the children were told that they could write the way they wanted and that it did not have to be like adults’ writing.

## The current study

In the current study we addressed the following research questions: (a) Does a 10-week invented writing program for five-year-olds carried out during the last term in preschool influence their phoneme awareness, spelling and word reading skills in preschool? (b) To what extent does an intervention effect in preschool affect early spelling and reading skills in school?

The invented writing program focused on children’s explorations of sound-text relationships during writing, for example writing their own names, shopping lists, messages and letters. The intervention was conducted in the Norwegian language, which has a semi-transparent phoneme–grapheme correspondence (Hagtvet, Helland, & Lyster, 2006; Seymour, Aro, & Erskine, 2003). The impact that the regularity of the orthographic system has on a child’s invented writing is not well understood. However, generalizing from studies of reading (Landerl, Wimmer, & Frith, 1997; Seymour et al., 2003) and from Martins et al. (2013) study of spelling, we would presume that it is easier for Norwegian speaking children to link phoneme to grapheme than for children making use of a deep orthography such as English. For example, in Norwegian the phoneme/a/would be pronounced similarly in *ball*, *katt*, and *land*, while *ball*, *hat*, and *garden* would be pronounced very differently in English (Furnes & Samuelsson, 2011). Therefore, the transparency of the orthography could have considerable impact on the results obtained from an invented writing intervention. Despite the overarching transparency in the Norwegian orthography, there are some stumbling blocks like consonant clusters (nifst; scary), doubling consonants (ball; ball), and one phoneme being represented by two or three letters (kj/tj, skj, and ng; Furnes & Samuelsson, 2011). For both theoretical and applied reasons, it is on this basis important to study the effects of invented writing intervention in different orthographies.

In the control group the children enrolled in a regular Norwegian preschool program participated. Although preschool is not compulsory in Norway, most children do attend (between 1 and 5 years: 90 % attendance; Statistics Norway, 2015). The preschools are often organized in mixed age groups, typically one-to-three and three-to-five year-olds. Although preschools are under the authority of the same ministry as schools, the educational philosophies vary. The preschool curriculum (The Framework Plan) has no specific learning aims. As is also the case in the other Nordic countries the children’s free play is highly prioritized together with outdoor activities, social skills and oral language acquisition (Norwegian Ministry of Education and Research, 2011). The children are not taught to read and spell in preschool, but the Framework Plan states that children should be familiarized with script symbols such as numbers and letters, and those who take initiatives to write should be supported by the preschool teacher (Norwegian Ministry of Education and Research, 2011). However, the plan offers no advice what to do with the children who do not take such initiative. One might worry that children who for various reasons do not take an interest in literacy activities will not get the input needed to get started with their early literacy learning in preschool. On this background, a major reason for developing the current program was to develop a child centered invented writing program that would potentially facilitate the literacy development of *all* children. The current program expands the invented spelling programs by including a broader set of writing activities (not only spelling) and also by encouraging the children to experiment with writing through dialogue with more competent peers and preschool teachers within a socio-cultural paradigm. By using this mediation procedure, the early literacy stimulation was carried out in ways which the teachers felt at home with and believed in (Vygotsky, 1978), and by carrying out an invented writing intervention program by the help of the children’s teachers, we could investigate its external validity with reference to a field experiment.

In summary, we wanted to investigate the effects of a preschool invented writing program on literacy skills in preschool (immediate post-test) and also on the subsequent skills of word spelling and word reading after 6 months in first grade (follow-up). The effect of the intervention was evaluated by comparing the children’s pre-test, post-test and follow-up performances. We hypothesized that the broader invented writing program where the children explored writing would have an immediate positive influence not only on the interconnected skills, phoneme awareness and spelling, but also on word reading skills. Moreover, if the invented writing program enhanced the children’s emergent literacy skills at post-test level, we expected this skill enhancement to facilitate formal learning to read in school. Given that the children started school between the post-test and the follow-up, we expected that an immediate direct effect of the invented writing intervention would level off as all the children were then introduced to reading and writing.

## Method

The current study employed a quasi-experimental pre-test post-test design to evaluate the effect of the intervention. The study was approved by the Norwegian Social Science Data Services. The twelve preschools were randomly assigned to one of two groups: an experimental condition with invented writing program (*n* = 40) or a control condition with the ordinary preschool program (*n* = 65). At the start of the intervention, the two groups showed no significant differences with respect to parental education level, gender, family size, home language, or the amount of shared book reading time at home, according to parents’ reports. The parents were generally well educated; 63 % of the mothers and 43 % of the fathers had a college/university education.

### Participants

All five-year old children in 12 preschools on the west coast of Norway were invited to participate in the study, and over 80 % of the parents accepted the invitation. Parental consent was initially given for 113 children; two children did not meet the inclusion criteria of speaking Norwegian well enough to be tested and were excluded. During the intervention period, six children (three from the intervention group and three from the control group) were excluded as they only participated marginally in the program (i.e., left preschool, went on vacation or moved), leaving the actual sample to 105 children (mean age = 5.7 years at pre-test; 54 girls and 51 boys). In the final sample, 11 of the included children were not native language speakers (their native languages included Somali, Polish, Dutch, and Romanian).

### Measures

#### Phoneme awareness

This study used three subtests from the Norwegian edition of The Comprehensive Test of Phonological Processing (Furnes & Samuelsson, 2009; Wagner, Torgesen, & Rashotte, 1999): sound matching, blending, and elision. During the sound-matching task, children are asked to point to a picture starting or ending with the same sound as a target word. During the blending task, children listen to words presented orally phoneme by phoneme and are then asked to tell what the intended word was. During the elision task, children are asked to repeat a word presented orally and then to omit a syllable or phoneme, for example, to say *kopp* (cup) without saying the/k/. Each subtest contains 20 items.

#### Spelling

The spelling tests included the following: (1) words, including ten high-frequency real words (e.g., *mann*; man and *lampe*; lamp), (2) non-words, including four non-words, (e.g., *ig* and *sut*). This spelling test was originally an English test developed by Byrne and Fielding-Barnsley (1993), which was translated to Norwegian and used in a cross-linguistic study by Furnes and Samuelsson (2009). In addition to this spelling test, we used an orthographic spelling test in first grade, including 11 words that were either rather long or orthographically challenging; mystisk (mysterious), elektrisk (eletric), klimpre (strum), klatrestativ (climbing frame), godt (well), opp (up), gikk (went), beskjed (notice), gjemsel (hide and seek), forsiktig (careful), vemmelig (nasty). In all the spelling tasks, the children were asked to write down the target words to the best of their ability. Each of the words was scored on a scale ranging from 0 to 6 points following the developmental scoring system in Byrne and Fielding-Barnsley (1993):

- No spelling attempt, or the spelling is not associated with any correct letter (score 0);
- One letter in the spelling is associated with one letter in the target word (score 1; e.g., child writes D for T in tog; train);
- One phoneme in the target word is correctly represented by a letter (score 2; e.g., child writes T for tog; train);
- At least two (but not all) phonemes in the target word are represented by a letter (score 3; e.g., TG for tog; train);
- All phonemes in the target word are represented in the child’s written product but not necessarily with the orthographically correct letters (score 4; e.g., TÅK for tog; train);
- All phonemes in the target word are represented by letters, but one phoneme is represented by a related letter that is not orthographically correct (score 5; e.g., TÅG for tog; train); or
- The spelling of the entire word is orthographically correct (score 6).

#### Sentence writing (only in preschool)

The sentence-writing task consisted of two words: *NN* (child’s name) *LIKER IS* (NN likes ice-cream). This task was included because it revealed the children’s spelling strategy quite clearly; many who did not write much succeeded in writing *IS,* but struggled with *LIKER.* Only the two words, and not the name, were scored. The children’s attempts to write the sentence were scored as follows: no attempt at writing (0), pre-phonemic spelling/scribbling (1), semi-phonemic spelling (less than 25 % of the letters correctly spelled; (2), advanced semi-phonemic spelling (25–75 % of the letters correctly spelled; (3), phonemic spelling (more than 75 % correctly spelled); (4), and correct spelling (5) (Child, Language and Learning, 2010).

#### Word reading

The children’s reading skills were measured using a Norwegian adapted edition of the Test of Word Reading Efficiency (TOWRE; Torgesen, Wagner, & Rashotte, 1999). The test included both a list with real words and a list with non-words. The children were asked to read as many words as possible from each list within 45 s.

#### Receptive vocabulary measure (pre-test only)

The children’s receptive vocabulary skills were measured using the Norwegian edition of the British Picture Vocabulary Scale II (Dunn, Dunn, Whetton, & Burley, 1997). The children were asked to select one of four pictures that best illustrated the meaning of the target word that was presented orally by the tester. The words represented a range of content areas, such as animals, actions, or emotions, with varying levels of difficulty. This variable was used as a control for differences between the two groups.

#### Letter knowledge

Children were presented with a paper containing 24 capital letters in a random order (excluding C, X, Z, Q, and W, but including the Norwegian letters Æ, Ø, Å). The children were asked to provide the letter sounds or letter names associated with the letters, and 1 point was given for each correct letter sound or name. This variable was included to check for pre-test differences between the two groups.

### Procedure

This study was conducted in four phases. First, the participating children were individually pre-tested in a separate room at their preschool (age five). Second, the children in the experimental group participated in the 10-week intervention program. Third, all children were individually tested at their preschools immediately following the intervention (i.e., in May/June, approximately 8 weeks prior to starting school in mid-August, age six). Fourth, all children participated in delayed post-test assessments 6 months later, (i.e., in November/December in grade 1, following 4 months of formally learning to read and write). The tests were administered by the first author or by trained research assistants. The testing time varied from 20 to 45 min per child.

#### The intervention program

In the intervention group, the children participated in group sessions 4 days per week for 10 weeks (with a total of 40 sessions scheduled). Each session lasted approximately 20 min and was conducted in small groups of four to seven children, depending on the number of children participating in each preschool. The program was conducted by the children’s teachers. Under the supervision of these teachers, the first 20 sessions were carried out by student teachers (i.e., students of a preschool teacher training program at bachelor level). Both the teachers and student teachers completed 2 h of training on how to administer the program prior to the start of the intervention, and a checklist detailing the session structure and keywords for scaffolding strategies was provided.

The teachers were trained to assess the children’s writing according to four development phases (pre-phonemic, semi-phonemic, phonemic and conventional; Hagtvet, 2010). The teacher supported the children’s writing at the individual child’s level. For a child at the pre-phonemic or semi-phonemic writing level, the teacher might for example encourage him or her to try to pretend write, or try to identify the first sound of the word. For a child writing at the phonemic level, the teacher might encourage him or her to identify the sounds in the word and to use letters to represent them. These mediation strategies (Vygotsky, 1978) by which the adult supported the children’s exploration of the oral-text connections were adapted to the writing level of the child. To maintain engagement and motivation, the teachers always praised some aspect of the child’s writing attempts, emphasizing that his or her writing did not need to be the same as adults’. I addition, the teachers explicitly supported the child through his or her challenges, for example with identifying the phonemes in words. In the group activities, the teachers referred to the phonemic segments of the target word to be written along with the children. For example, the children were asked to say the target word slowly and to listen actively as they wrote the word to identify what sounds they could hear. The children were also asked to help each other, for example: “Ask Lisa if she could help you with the letter L because that is the first letter in her name, Llll-isa.” By this approach the children were made aware of the phonemes in words in connection with their own writing of the words and in their individual pace.

Letter names were not systematically taught, but a capital letter alphabet was provided on a sheet or on the wall in the classroom and was used for children to copy during the writing activities. By this approach the letters were learned as they were needed during the writing of words; they were learned as linguistically functional entities (Frost, 2001) in the sense that they were used to make words. An inbuilt progress in the presentation of words used for writing made certain that the children had to deal with most of the letters of the alphabet during the program.

The program consisted of two parts (see detailed program in the appendix). Part I was conducted during the first 3 days of each week (30 sessions) and included three activities:

1. The children generated new words from a list of two-letter prompts by adding letters; for example, LE became LES when adding/s/to/le/. The adult wrote the children’s suggestions on a flip chart.
2. The word of the day was written on a flip chart, such as the name of one child in the group or a short word such as HUS (house) or BÅT (boat). By letting the children write and explore each other’s names we wanted to make the task meaningful for the children (Bloodgood, 1999). The word was discussed in the group using phrases such as: “What sounds can we hear in this word?”, “Is it a long or a short word?” and “Do we know any of the letters?” Besides supporting the children’s phoneme awareness, in these two activities the children were introduced to the writing process; they were shown what we do and think when writing and also allowed to practice to write the words with mediation support from the teacher.
3. Provided with a notebook, scissors and pictures on a sheet, the children were asked to cut out a picture chosen freely (e.g., animal, cartoon or toy), paste it into the notebook, and write down what was on the picture. The child’s task was now to make use of his/her knowledge about writing when independently writing the words of his/her choice. The children received individual feedback from the teachers on the word they were writing; first, the child read aloud what he or she had written. Then the teachers praised the child’s spelling relative to his or her level of writing, and wrote in conventional orthography what the child had read aloud, but without commenting on the child’s writing. The teachers’ writing provided alphabetic feedback to the children; it was given in the group of children to encourage learning from each other’s feedback.

Part II of the program (10 sessions) was administered one day each week and consisted of an explorative and inventive writing session. The purpose was to encourage and support independent invented writing by for example writing a shopping list, cards or a note. As before, the children were instructed to write the words in their own way and ensured that their writing did not need to be identical to adults’ writing. The children were also encouraged to help each other segmenting words into phonemes and demonstrate letters to each other. At the end, the children read aloud what they had written, the teacher praised the children’s writing and delivered a model of convention writing, for example; “Oh, look how much you have written, Marie! It looks exciting! Shall we try to read it?” The teacher then provided individual feedback by writing in conventional orthography what the child had read.

#### The control group

The control group consisted of peer-aged children who followed the ordinary preschool program offered in their communities. These were typical Norwegian preschool education programs focusing on oral language, social skills and shared book reading. The children were actively involved in outdoor activities and free playing of their own choice. Shared book reading is a favored activity in all Norwegian preschools, many preschools have oral phonemic awareness training, a number of children always write spontaneously and some children are taught letters and reading/writing at home. The control children participated at least once a week in special groups for five-year-olds that emphasized the learning of rhymes, dialogic reading, game playing, storytelling, drawing, and writing their own name on their drawings.

### Intervention fidelity

Teacher logs were used to assess the fidelity of each intervention session, including the extent to which the delivery of an intervention program is conducted as designed in terms of both the dosage and the content (Swanson, Wanzek, Haring, Ciullo, & McCulley, 2013). The teacher logs included attendance and completed activities of each session. In addition to the logs, the first author visited all preschools to observe and to make sure any clarification needed was given before and during the intervention period. These observations indicated that the intervention program was followed. The intended duration and dosage of the program was 40 sessions lasting 20 min each, but the teacher logs revealed that the average number of sessions was 36. The content of the program was assessed using a checklist included in the daily teacher logs. The checklist responses were filled out using a Likert scale ranging from 1 (low correspondence) to 5 (high correspondence). The content fidelity percentages were calculated by comparing the correspondence between the program as designed and as administered. The overall content fidelity was high, with 98 % of sessions administered in accordance with the plans; however, there were examples of deviancies, such as skipping the two-letter prompts, or even replacing activities with others.

### Analytical strategy

We used structural equation modeling (SEM) with Mplus 7 (Muthén & Muthén, 2012), using autoregressive models to examine the latent variables at the pre- and post-test stages. SEM was preferred as statistical technique over repeated measure ANOVA because it has several strengths such as the ability to correct for bias in estimates of standard errors due to cluster randomization, as well the ability to analyze several equations simultaneously and to estimate indirect effects (Tabacknick & Fidell, 2013). The analyses used multiple observed indicators of each target latent variable; hence the common variance of the construct indicators was analyzed, thereby avoiding bias due to errors of measurement. To control for pre-test differences and to reduce the influence of low variance in the reading and spelling variables (many of the children could not read or spell and therefore scored zero), we used longitudinal latent variable models with multiple indicators. One dummy variable was used to represent the two groups; the control group was used as the reference group and coded zero. Robust maximum likelihood estimation (MLR) was used, and the effect sizes (*d* values) for the intervention were calculated based on the partial regression coefficients for the dummy variable.

The participating 105 children were nested in 12 preschools. To check the level of difference due to which preschool the children attended, we measured the intra-class correlation coefficient (ICC). An ICC with a value of 1 would indicate that all variance was at the preschool level (i.e., the responses from children in the same school were identical; hence, the effective sample would be 12). An ICC with the value of 0 would indicate that the children’s responses were uncorrelated (i.e., the variance between the children was independent of preschool affiliation, and the effective sample would be the same as the observed sample of 105). The analysis showed ICC values ranging from .04 to .19 for the pre-tests: letter knowledge (i.e., vowels = .19 and consonants = .18), vocabulary (=.17), reading (i.e., words = .15 and non-words = .12), spelling (i.e., words = .13, non-words = .14), and phoneme awareness (i.e., blending = .16, elision = .16, sound matching = .04). Although the ICCs were modest, suggesting that the literacy skills among the children attending different preschools were similar, they were large enough to indicate that the randomization at the preschool level implied a loss of statistical power (Hox, 2010). The results showed that there were small differences between preschools in sound matching, and larger differences in letter knowledge and vocabulary. Thus for letter knowledge and vocabulary, which preschool had been attended accounted for 19 and 17 % of the variance respectively. To prevent bias due to intra-class correlation, cluster-robust standard errors were estimated in all analyses using the so-called “complex option” in Mplus, which controls for effects of preschool nesting on the standard errors.

The three latent variable models for spelling, phoneme awareness and reading development were considered separately because of the modest sample size. A regression on the group dummy was conducted to estimate the immediate intervention effect and the direct and indirect effects of the intervention on the follow-up test. The model fit and the direct and indirect effects of the program on the children’s literacy skills as assessed in preschool and in school are presented for each of the three models. The model fit was indicated by Chi square test statistics and goodness of fit indices. As a rule of thumb, good fit could be indicated by a ratio of the Chi square to the degree of freedom under two (Tabacknick & Fidell, 2013). Furthermore, an acceptable model fit could be indicated by a root mean square error of approximation (RMSEA) value below .08, comparative fit index (CFI) and Tucker-Lewis index (TLI) values above .95, and a standardized root mean square residual (SRMS) value below .08 (Kline, 2011).

## Results

### Pre-test, post-test and follow-up measures

Descriptive statistics with the means and standard deviations for both groups at the pre-test, post-test and follow-up are shown in Table 1, including the reliabilities of the measures at pre-test (Cronbach’s alpha). Levene’s test for equality of variance revealed no significant group differences for any of the pre-test measures. At pre-test level, some of the children could spell a few words, the mean letter knowledge was 12–13 letters, and they were able to determine the first sounds in words by listening. Only 17 % of the children could read words. The table shows the children’s performance immediately after the interventions (post-test) and 6 months later in school (follow-up). The children who were given the invented writing program had higher mean scores for all post-test and follow-up measures of spelling, phoneme awareness, and word reading than the children in the control group. Most interesting, at the follow-up test 6 months later, the children in the invented writing group spelled more orthographically challenging words correctly.

**Table 1:** Pre-test, post-test, and follow-up literacy measures: means, standard deviations and reliabilities

Measure (maximum)	Invented writing group (n = 40)			Control preschool group (n = 65)			α
	Pre-test	Post-test	Follow-up	Pre-test	Post-test	Follow-up	
*Spelling*							
Words (60)	10.70 (19.50)	28.92 (21.13)	47.20 (14.21)	11.42 (18.20)	15.98 (20.51)	39.05 (19.29)	.979
Non-words (24)	3.78 (7.52)	10.35 (10.01)	18.85 (5.70)	3.80 (7.10)	5.49 (8.32)	14.37 (8.49)	.957
Sentence (5)	2.47 (1.52)	3.58 (1.17)	–	1.92 (1.78)	2.28 (1.71)	–	
Orthographic spelling (66)		–	40.08 (14.20)	–	–	31.15 (17.22)	
*Phoneme awareness*							
Sound matching (20)	5.65 (4.17)	8.63 (5.49)	13.97 (5.68)	6.18 (4.83)	6.77 (4.99)	10.83 (5.66)	.898
Blending (20)	4.30 (3.70)	7.58 (4.72)	12.50 (3.67)	4.17 (3.76)	5.43 (4.57)	10.55 (4.72)	.887
Elision (20)	2.28 (1.45)	4.35 (2.42)	6.25 (2.96)	1.69 (1.45)	3.03 (2.89)	5.00 (3.40)	.615
*Word reading*							
TOWRE words	1.10 (3.02)	3.35 (5.15)	12.25 (8.88)	1.18 (3.14)	1.63 (4.00)	8.88 (10.13)	.937
TOWRE non-words	1.15 (2.94)	3.63 (4.99)	10.25 (6.86)	1.00 (2.60)	1.48 (3.62)	7.00 (7.70)	.920
Vocabulary (144)	54.75 (10.06)			54.85 (10.78)			.908
Letter knowledge (24)	13.18 (7.08)	17.53 (6.72)	–	12.34 (8.29)	13.80 (7.61)	–	

α = Cronbach’s alpha

The correlations between the variables at pre-test, post-test and follow-up levels are shown in Table 2. The emergent literacy variables were highly correlated, with the exception of vocabulary knowledge and reading at pre-test. There were high correlations between the pre-test spelling and reading and high correlations between concurrent phoneme awareness and spelling at all three time points.

**Table 2:** Correlations between the emergent literacy variables across all three time points

	1	2	3	4	5	6	7	8	9	10
1. Spelling T1	–									
2. Reading T1	.80**	–								
3. Phoneme awareness T1	.74**	.67**	–							
4. Vocabulary T1	.28**	.18	.30**	–						
5. Spelling T2	.71**	.59**	.63**	.21*	–					
6. Reading T2	.67**	.72**	.61**	.21*	.67**	–				
7. Phoneme awareness T2	.71**	.60**	.75**	.30**	.77**	.76**	–			
8. Spelling T3	.44**	.30**	.50**	.64**	.64**	.42**	.61**	–		
9. Orthographic spelling T3	.47**	.33**	.52**	.28**	.65**	.47**	.64**	.95**	–	
10. Reading T3	.60**	.48**	.56**	.28**	.69**	.71**	.70**	.69**	.72**	–
11. Phoneme awareness T3	.57**	.46**	.63**	.33**	.71**	.60**	.73**	.86**	.84**	.79**

Spelling = a composite of spelling words and spelling non-words; reading = a composite of TOWRE words and TOWRE non-words; phoneme awareness = a composite of sound matching, blending and elision; and T1 = pre-test, T2 = post-test, and T3 = follow-up

* *p* < .05; ** *p* < .01

**Fig. 1:**
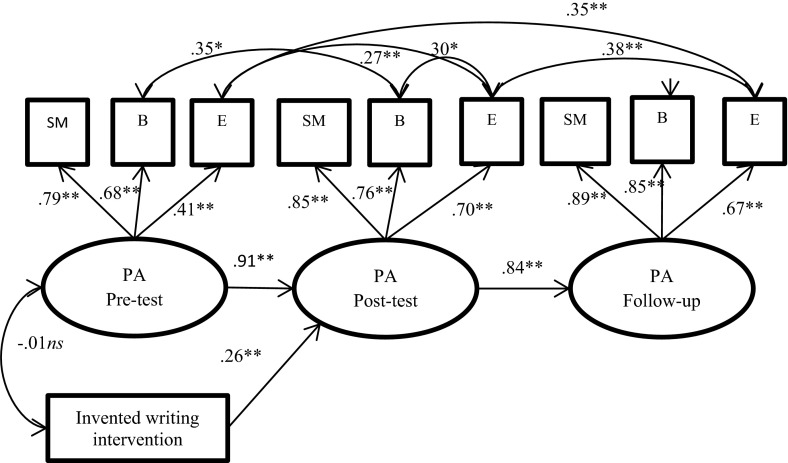
Path diagram of the longitudinal effects of the intervention on phoneme awareness at post-test and follow-up in school, with a dummy for the intervention group and with the control preschool group used as a reference group. Standardized coefficients are presented. *PA* phoneme awareness, *SM* sound matching, *B* blending, and *E* elision. **p < .01; *p < .05, *ns* not statistically significant

**Fig. 2:**
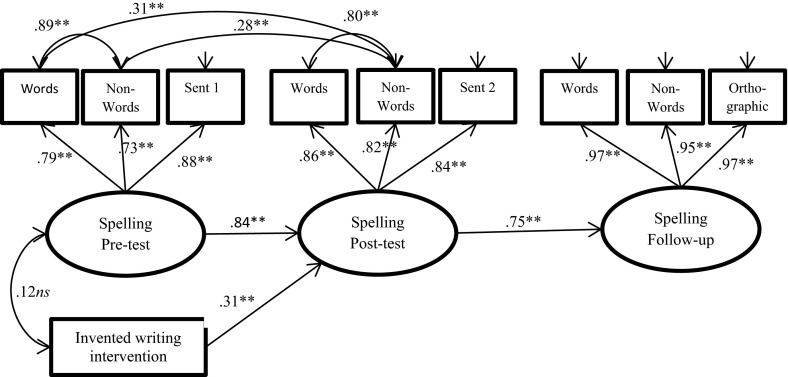
Path diagram of the longitudinal effects of the intervention on spelling at post-test and follow-up in school, with a dummy for the intervention group and with the control preschool group used as a reference group. Standardized coefficients are presented. *Sent* sentence task. **p < .01; *p < .05, *ns* not statistically significant

**Fig. 3:**
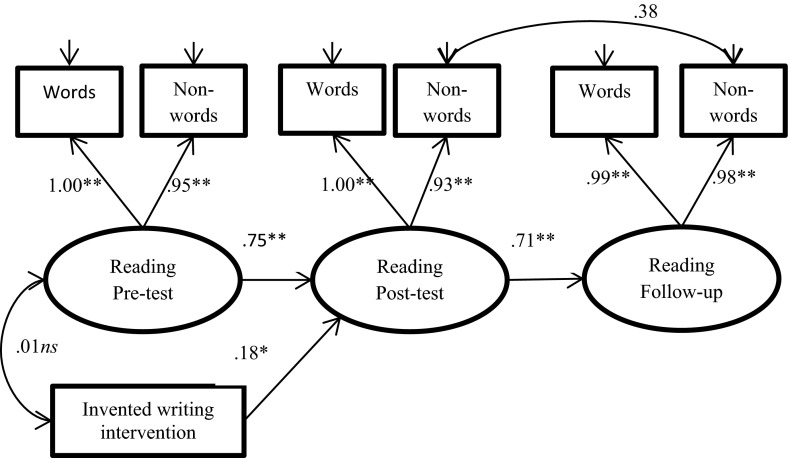
Path diagram of the longitudinal effects of the intervention on reading at post-test and follow-up in school, with a dummy for the intervention group and with the control preschool group used as a reference group. Standardized coefficients are presented. **p < .01; *p < .05, *ns* not statistically significant

To assess the effects of the invented writing program, we used autoregressive latent variable models for the constructs of phoneme awareness, spelling and word reading. Latent variables allowed us to combine different types of observed variables to measure the hypothetical constructs of phoneme awareness, spelling and reading (Kline, 2011). In our analysis, the latent variable for phoneme awareness had three indicators (i.e., sound matching, blending and elision) in the pre- and post-tests (see Fig. 1). The latent variables for spelling had three indicators (i.e., words, non-words and sentence writing) at the pre- and post-tests, in addition to an orthographic spelling indicator at the follow-up stage in school (see Fig. 2). The latent variable for word reading had two indicators (i.e., TOWRE words and TOWRE non-words) at all three testing points (see Fig. 3). The residuals of corresponding indicators could be correlated across the three time points. To evaluate the intervention effects, a dummy variable for the intervention group was regressed on the latent variables of the post-test and follow-up scores, controlling for the pre-test score. The three autoregressive models showed acceptable goodness-of-fit statistics (see Table 3).

**Table 3:** Goodness-of-fit statistics for the autoregressive latent variable models

Model	χ^2^ (*df*)	χ^2^/*df*	CFI	TLI	RMSEA	SRMR
Phoneme awareness	28.89 (26)	1.11	.994	.990	.033	.047
Spelling	58.63 (30)	1.95	.979	.968	.095	.040
Word reading	10.52 (8)	1.32	.995	.987	.055	.028

χ^2^ Chi squared; *df* degree of freedom, *CFI* comparative fit index, *TLI* Tucker–Lewis index, *RMSEA* root mean square error of approximation, *SRMS* standardized root mean square residual

* *p* < .05

The path diagram for the phoneme awareness model is shown in Fig. 1, that for spelling in Fig. 2, and that for word reading in Fig. 3. As presented in Fig. 1, the estimated standardized factor loadings for phoneme awareness ranged from .41 to .89, with the lowest factor loading for elision at the pre-test stage. This result suggests that the task was difficult for the children at this point in time. There were significant correlations among the residuals for corresponding measures across time, ranging from .38 to .27. The factor loadings for spelling ranged from .73 to .97 (Fig. 2). The residuals for words and non-words were highly correlated (*r* = .89 at pre-test and *r* = .80 at post-test), suggesting that spelling words and spelling non-words were nearly the same task at the beginner spelling level. The factor loadings for word reading were high, ranging from .93 to 1.00 (Fig. 3). The autoregressive relations among the latent variables (i.e., phoneme awareness, spelling, and word reading) at post-test and follow-up level revealed high levels of longitudinal stability in all three models.

In Table 4, the effects of the intervention become apparent. Invented writing program had a significant immediate direct effect on phoneme awareness, spelling and word reading in preschool. The effect sizes were moderate for all variables, with larger effect sizes for phoneme awareness (*d* = .54) and spelling (*d* = .65) than for word reading (*d* = .36). At the follow-up in grade 1, lasting indirect effects of the invented writing program were observed on phoneme awareness, spelling and word reading, mediated by the post-test variables (Table 4).

**Table 4:** Effects of the invented writing intervention

Outcomes	Post-test in preschool (age 5) (direct effects)	Follow-up in grade 1 (age 6) (indirect effects)
*Invented writing group*		
Phoneme awareness	.54** (*t* = 4.37)	.45** (*t* = 4.40)
Spelling	.65** (*t* = 4.48)	.48** (*t* = 4.61)
Word reading	.36* (*t* = 2.51)	.26* (*t* = 2.53)

* *p* < .05; ** *p* < .01

To summarize, the invented writing program showed significant effects of moderate sizes for spelling, word reading and phoneme awareness immediately following the intervention. In grade 1, the effects of the preschool invented writing program were still present, but they were smaller and indirect.

## Discussion

In the current study, we evaluated the effects of a 10-week invented writing program on phoneme awareness, spelling and reading skills in a semi-consistent orthography. The immediate and delayed effects of the intervention program were assessed. As expected, the results from the immediate post-tests revealed significantly improved phoneme awareness, spelling and word-reading skills for the invented writing group compared with the control group. Following the lexical quality hypothesis (Perfetti & Hart, 2002), in the process of writing words and texts; phonemes, graphemes and semantic content are handled as interactive entities where each entity presumably reinforces the others. Notably, these results were obtained in a field experiment that was conducted in natural preschool settings by the children’s teachers, whereas the comparable intervention studies were conducted by researchers. These results were obtained despite the fact that fidelity investigations revealed that the number of intervention sessions was lower than intended (36 rather than 40 sessions) and that the program was more child-driven and explorative, focusing on invented *writing,* than the comparable previous studies which focused on *training of spelling*. Taken together this indicates that the reading development of these children benefited from an invented writing program carried out in a relatively short period of time where children’s invention and exploring of phoneme-grapheme connections were crucial. Theoretically, these findings corroborate research that highlights invented writing as a particularly powerful way of strengthening a child’s phonemic awareness and subsequent word decoding skills. They also invite reflections on the relative ease with which children in general appear to learn to read and spell alphabetically within a semi-consistent orthography like the Norwegian (Landerl et al., 1997).

The limited research available on interventions with invented spelling in preschool has been carried out in English, French, Hebrew and Portuguese orthographies. The current observation of a significant effect of the invented writing program on preschool novel word reading is consistent with studies of Portuguese speaking children (Martins et al., 2013, 2014). None of the previous studies with deep orthographies, like English and French, observed transfer effects to new word reading. Taken together these findings suggest that with a semi-transparent orthography it is easier for a child to come to terms with the alphabetic principle; it apparently also simplifies the transfer from practicing invented spelling to reading. Martins et al. (2014) found a larger effect size (Cohen’s *d* = 1.06) for word reading than observed in the current study. This may reflect program differences, for example that 13 of the 26 words the children were asked to read in the Portuguese study consisted of consonants that were practiced in the program, while the letter knowledge was integrated in the writing activity in the current program, suggesting that the children learned the letter names while using them during writing. Despite these and other differences, for example in feedback procedures, both these studies, conducted in fairly consistent orthographies revealed transfer effects from children’s exercising of writing/spelling to their word decoding, while the studies with French and English orthographies did not. The studies are small in both size and number, and generalizing conclusions must therefore be drawn with care, but the findings suggest that a more consistent orthography appears to facilitate not only the breaking of the alphabetic code, but also the transfer from invented spelling to novel word decoding.

The follow-up test furthermore revealed continuing indirect effects of the invented writing intervention in the current study on phoneme awareness, spelling and reading in grade 1, suggesting that the invented writing program in preschool positively supported the children’s literacy learning in school. This finding corroborates Ouellette et al. (2013) observation of delayed effects of invented spelling training on spelling skills 6 months after the intervention. In addition the current study showed delayed transfer effects to word reading. These transfer effects suggest that the general literacy skill enhancement observed at post-test facilitated the more formally taught reading and spelling skills in grade 1. However, it must be noted that the descriptive mean differences between the invented writing group and the control group were not large, and the size of the effect on phoneme awareness, spelling and word reading in grade 1 was also relatively modest. This was in accordance with expectation, given that the children started school between the post-test and the follow-up. During this period all the children were introduced to reading and spelling, and it was to be expected that an immediate direct effect of the invented writing intervention would level off. Schooling has a homogenizing effect on children’s reading and effect sizes therefore tend to diminish over time in reading intervention research (Fletcher & Wagner, 2014). Hence, smaller indirect effect sizes should therefore not be interpreted as indicating that “invented writing” had lost its long-term predictive power, but rather be seen as important indicators of the participating children’s accumulated knowledge about literacy. They may result in major changes in future performance if they continue to cumulate. Several studies have shown that children’s reading ability in grade 1 predicts their subsequent reading levels (see Cunningham & Stanovich, 1997; National Early Literacy Panel, 2008; Verhoeven & Leeuwe, 2008). On this backdrop our findings suggest that invented writing could be a useful tool for supporting children’s early literacy learning including reading skills.

## Cautions and limitations

This study was limited by the modest sample size and by randomization at the preschool level. We used cluster-robust estimates of standard errors, but randomization at the cluster level inevitably implies a loss of statistical power. Additionally, a floor effect was observed in the pre-test measures for word reading and spelling because the majority of the children were pre-literate. This effect resulted in low levels of variability in the word reading and spelling variables. Overall, despite these methodological limitations, this study has shown that a preschool-based invented writing program may benefit children in their development of emergent literacy skills. However, due to a small sample size and no control intervention, generalizing conclusions should be made with care.

The preschool teachers were used as instructors in the intervention; we cannot therefore exclude a trade off from their interaction with the children during writing in the program sessions to interactions during the rest of the days to the effect that the current program was more extensive than described. However, there are no indications to that effect. On the contrary, with the Norwegian focus on the importance of outdoor activities, free play and social skills, the indications are that the teachers made an effort to balance the program off with these other activities. The positive findings were observed in a semi-transparent orthography. This invites studies that compare interventions in transparent and deep orthographies to clarify how effects differ across orthographies.

The control group received a program that was typically offered to five-year-olds in their local area; this is in general considered a good program (Engel, Barnett, Anders, & Taguma, 2015). Both for substantive and ethical reasons it would have been advantageous to treat the controls as a waiting list control group. However, the intervention took place during the last term in preschool and time restrictions therefore excluded this design. The control preschool teachers were offered the invented writing intervention program together with a lecture about the program after positive effects had been documented and they could therefore use it in their subsequent preschool practice.

## Conclusion

The current study addressed the following research questions: (a) Does a 10-week invented writing program for five-year-olds carried out during the last term in preschool influence their phoneme awareness, spelling and word reading skills in preschool? (b) To what extent does an intervention effect in preschool affect early spelling and reading skills in school? Our findings were encouraging: the invented writing program implemented in a natural classroom-based setting increased the children’s beginning spelling and reading skills as assessed immediately after intervention; it also had a positive effect on phoneme awareness, which is an important foundation skill for learning to read. Finally, the program seems to have a positive indirect effect on phonemic awareness, spelling and word reading midway through grade 1; therefore, we argue that the intervention made literacy learning easier for the children. This includes learning to decode words. These findings have theoretical and applied implications. The process of translating from spoken to written language during exploratory writing appears to make children phonemically aware while at the same time expanding their knowledge of the alphabetic code at a functional level where phonemes and graphemes are continuously used to create meaning. Our results suggest that these assets of the writing process presumably make invented writing a powerful entrance to literacy maybe in particular in a fairly consistent orthography, where the transformation from sounds to letters is relatively straight forward.

## Appendix

Invented writing program

Part I:

Invented writing activities used on the first 3 days each week (30 sessions)

- Group activity: Writing words of a two letter prompts; week 1: IS, week 2: LE, week 3: AL, week 4: EL, week 5: SI, week 6: OL, week 7: NI, week 8: GA, week 9: MØ, week 10: KU
- Group activity: Writing the word of today, examples of target words used in the second activities: Names of the children in preschool + hus (house), lus (lice), mus (mouse), brus (soda), ski (skii), skilt (sign), bil (car), bilde (picture), båt (boat), tog (train), fly (air plane), katt (cat), kake (cake)
- Invented writing individually, freely chosen writing activity with pictures of toys, cars, animals, etc.

Part II:

Invented writing activities used on the fourth day each week (10 sessions):

*Week 1. Shopping list:* children wrote and draw shopping list

*Week 2. Note to parents:* children wrote a reminder for example extra mittens or fruit.

*Week 3. This makes me happy/sad:* children draw pictures of the theme and then wrote what they have been drawing.

*Week 4. Finding logos in preschool*: the children were seeking for logos in the classroom and then copied the logos by writing them on sheets

*Week 5. Card:* children wrote a card to a friend.

*Week 6. Shopping list*

*Week 7. Note to parents*

*Week 8. My favorite food:* drawing pictures of food and writing

*Week 9. I like to do:* drawing pictures of the theme and then writing

*Week 10. Card*
